# Dental Identification System in Public Health: Innovations and Ethical Challenges: A Narrative Review

**DOI:** 10.3390/healthcare12181828

**Published:** 2024-09-13

**Authors:** Gabriele Napoletano, Alessandra Putrino, Enrico Marinelli, Simona Zaami, Lina De Paola

**Affiliations:** 1 Department of Anatomical, Histological, Forensic and Orthopedic Sciences, Sapienza University of Rome, 00161 Rome, Italy; gabriele.napoletano@uniroma1.it (G.N.); lina.depaola@uniroma1.it (L.D.P.); 2 Department of Oral and Maxillofacial Sciences, “Sapienza” University of Rome, 00161 Rome, Italy; 3 Department of Medico-Surgical Sciences and Biotechnologies, Sapienza University of Rome, 04100 Latina, Italy

**Keywords:** dental identification systems, forensic identification, medical devices, public health, vulnerable populations, dentistry liability, military risk

## Abstract

Dental identification systems (DISs) encompass various techniques used for forensic identification, serving as alternatives or complements to genetic methods. Technologies such as microchip implants, prosthetic inscriptions, microSD cards, and identification plaques have been proposed to address limitations in comparative methods, offering streamlined processes for forensic experts. This study reviews current and potential DIS implementations, emphasizing cost-effectiveness and community benefits. Literature analysis from PubMed (2008–2024) yielded 17 relevant articles on implantable DISs, enabling direct subject identification via teeth or prostheses. The integration of DIS aims to enhance accuracy and speed in personal profiling and legal identification, promoting technology transfer in dentistry. It will be necessary to develop strict privacy regulations to protect patient data and establish ethical guidelines for their use. The study’s aim is to highlight that the universal adoption of DISs could mitigate healthcare disputes and facilitate data exchange in clinical settings, which is particularly beneficial for vulnerable populations.

## 1. Introduction

Methods aimed at personal identification present practical aspects of undoubted interest for both clinical dentistry and forensic dentistry. The study’s aim is to highlight that the universal adoption of DISs could mitigate healthcare disputes and facilitate data exchange in clinical settings, which is particularly beneficial for vulnerable populations.

Dental identification systems (DISs) encompass various techniques used for this purpose, sometimes in combination and other times as alternatives to genetic identification methods [1]. Being able to attribute an identity to an unidentified subject through dental procedures can have concrete utility and provide a solution to a problem that is simultaneously human and ethical, as well as administrative and legal [2]. The use of low-cost identification systems could be devised to provide detailed information about the subject carrying the DIS, as well as regarding the materials used, the hospital structure, and the professional who implemented it. Over the years, several DISs have been proposed, including the implantation of microdevices, inscriptions on prostheses, labels, alphanumeric codes, microSD cards, and identification plaques, primarily aimed at facilitating the identification of individuals involved in mass disasters [3]. 

In the process of identifying an unnamed body, fingerprinting is usually conducted, a biological sample is collected for DNA analysis, and a comparison of post-mortem findings with ante-mortem ones begins, which can be time-consuming and not always lead to a definite identity [4]. In mass disasters (terrorist attacks, plane or ship accidents, earthquakes, hurricanes, etc.), due to the high number of victims and the degree of destruction of their bodies, the recognition phases may require numerous human and financial resources. Moreover, in deaths occurring in large fires or in post-mortem combustion of human remains up to incineration, the chances of establishing a definite identity through these means are drastically reduced [5]. Sometimes, the same DNA investigations may prove futile due to sample denaturation. Dental identification systems (DISs), thanks to the thermoresistance of the materials with which they can be made, not only could offer the possibility of recognizing a charred or incinerated body but also enable it through a direct mechanism of accessing the information contained therein. Another element to consider is the time required to identify a large number of bodies. 

In fact, in disasters where the number of victims is very high, logistical and organizational capabilities can be limited, and the onset of decomposition phenomena in biological specimens often complicates the identification process [6]. In this case, as well, the presence of a DIS could streamline the work of forensic experts [7]. One of the most used dental identification systems is the alphanumeric system, which involves the use of letters or numbers with the assignment of an individual identification code, often already in use for other services. Each code is then associated with an identity that can be verified at any time. In some countries, marking medical devices, teeth, dental prostheses, and removable orthodontic appliances for identification purposes (e.g., with electronic devices, labels, laser marks, or QR codes) is a procedure mandated by law, sometimes funded by the state [8,9,10]. In the USA, 21 out of 50 states have mandated by law the marking of prostheses. In Europe, although the practice is recommended, there are no specific legal regulations explicitly requiring it for personal identification purposes [4]. In Italy, for example, any medical device, including dental prostheses, must be marked with a unique and traceable label according to Regulation (EU) 2017/745 (MDR), which came into force on 25 May 2017 [11]. Since its application date, the MDR has replaced the existing Directives on Medical Devices 93/42/EEC and on Active Implantable Medical Devices 90/385/EEC, which regulated the medical device sector within the EU [12]. The main innovation of this regulation is the establishment of the Unique Device Identifier (UDI) system for the unique identification of devices. 

The UDI—Unique Device Identifier—is represented by a series of numerical or alphanumeric characters created based on accepted device identification and coding standards internationally, enabling the unequivocal identification of specific devices on the market (Medical Devices Regulation art. 2, paragraph 15). The UDI system applies to all medical devices except for custom-made devices. Furthermore, concerning the MDR’s prescriptions on implantable medical devices, the guideline of the Medical Devices Coordination Group (MDCG) provides useful indications, including examples, on how the so-called implant passport should be [13]. 

Understanding who intervenes and how they intervene in the processes of managing and supplying medical devices (MD) does not allow us to directly connect an MD to a patient or healthcare provider, neither in cases of medical-legal disputes for professional liability nor in cases of identifying unidentified victims. Furthermore, as mentioned, the MDR is not considered for custom-made devices. This is why the concept of MD traceability must be more widely used, expanded, and perhaps integrated through dental identification systems (DISs). DISs can be produced using various methods and techniques, and numerous materials have been proposed for their implementation. The choice of an encoded informative microchip involves its insertion into a cavity drilled into the tooth and the subsequent filling of the cavity with fire-resistant material. 

This technology involves a reading phase by an operator who, with the help of a microprocessor, accesses the person’s information. In the case of labeling, a miniaturized identification code will be attached externally or internally to the prosthesis or tooth. More recently, laser marking systems have been used [14]. To produce these devices, the choice of materials is important as they must be both economical and resistant to various chemical and physical agents, such as acids and high temperatures [15,16]. At the same time, they must be materials that do not harm the individual’s health. In external fissures, it is important not to create excessively deep cavities that could lead to medical issues [17] or become sites for food and bacteria to accumulate. Conversely, with raised labeling, it is advisable to prefer surfaces that minimize excessive rubbing and irritation of the oral mucosa [14]. Sometimes, alongside the unique national code, the name of the receiving individual or other personal and/or medical information can be added [8]. Equally important is the inclusion of the names of the professionals who performed the work and the hospital facility where the medical procedure took place. This aspect not only demonstrates transparent communication between the user and the hospital but also provides assurance to the patient that in case of medical malpractice related to the implant insertion and implementation procedures, they can more easily assert their right to compensation without facing uncertainty regarding the attribution of the device.

## 2. Materials and Methods

**Figure 1:**
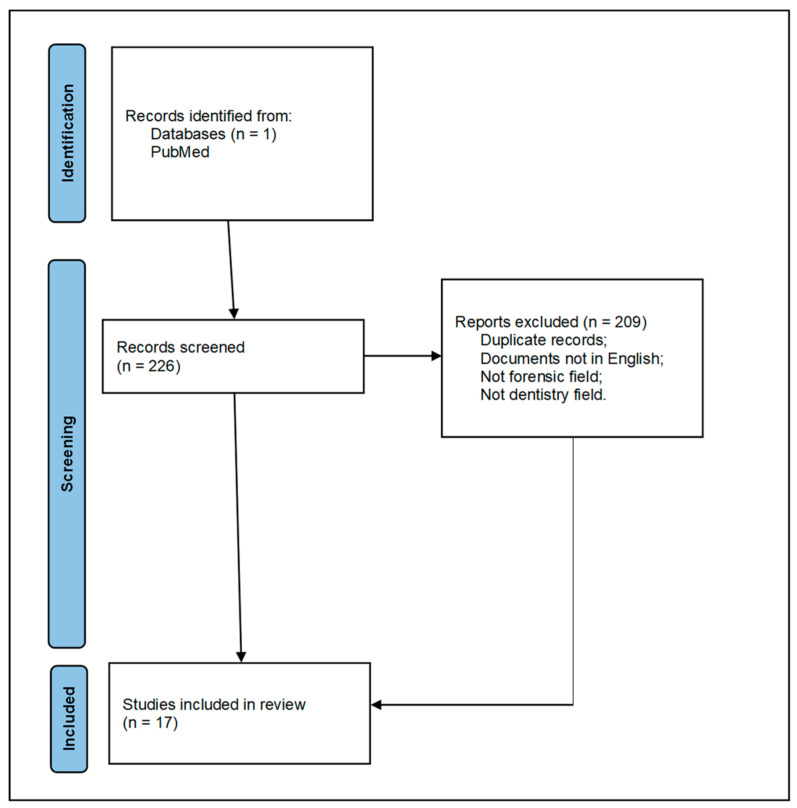
Prisma flow chart.

The present study analyzed articles available on PubMed over the past 16 years, from 2008 to 2024. Using the search terms “dental” AND “identification number” AND “forensic”, 226 results were found. Among these, duplicates and results dealing with forensic identification in general were excluded. Only articles in the English language were included. Ultimately, as summarized in Figure 1, 17 articles were included that described dental identification systems implantable on teeth or prostheses useful for directly determining the subject’s identity. This article aims to update a study conducted in 1993 [18], which proposed a dental identification system to be applied to a tooth subjected to conservative treatment. Although there is an international standard [19] for marking, the objective of this study is to provide an overview of the dental identification systems used without significant individual and social costs in order to evaluate their feasibility for widespread use.

## 3. Results

The importance of a universal and immediate dental identification system is supported by various authors (Table 1). Among the scientific works included in our study, 12 were conducted in India, indicating the widespread application of dental identification systems. The remaining studies report experiences shared by authors practicing in Saudi Arabia, Chile, Australia, the United States, and the United Kingdom. Most of the innovations proposed in this field originate from countries where the use of dental identification systems is regulated by law. 

The regulation of Dental Identification Systems (DIS) varies by country and is often integrated into broader forensic and health information regulations, and specific practices may vary widely between jurisdictions. Compliance with national data protection and privacy laws is essential in most countries, influencing how dental records are used and stored in forensic contexts.

The primary interest has been to provide certainty of identity to victims of mass disasters. This is crucial for the families of the victims and for the social, administrative, legal, and insurance implications that arise from it. In addition to the identification purpose—related to mass disasters (fires, hurricanes, cyclones, earthquakes, accidents, or terrorist attacks), dental identification systems have been used to expedite care processes for patients who are unconscious or unable to provide necessary health information, for example, due to a language barrier. 

One of the advantages of dental identification systems is their ability to store numerous personal data, including the patient’s medical history. Dental identification systems have been applied to hospitalized individuals, in care facilities, or at home with neurodegenerative diseases such as Alzheimer’s and Parkinson’s. These patients, often older and more frail, may not recall important health information and may experience confusion; they may sometimes wander off alone and even become lost in isolated and dangerous places. In all these cases, dental identification systems are strongly recommended. Table 1 summarizes various dental identification systems (DISs), including the country of origin, the type of DIS used, the material, the manufacturing method, and the type of patient for whom the DIS is applied.

## 4. Discussion

Dental identification systems (DISs) are extensively studied tools in forensic identification, particularly for determining the identity of an individual [35]. Unlike other methods of identification (DNA, radiographic examinations, or unique physical features), DISs provide immediate information about a subject’s identity without further evaluations or comparisons. This rapidity and certainty of results represent a significant advantage, especially in cases of mass disasters involving hundreds of victims. In such situations, expert personnel are required to promptly assign an identity to unidentified individuals before decomposition processes and the action of microorganisms make corpse examinations impractical. Dental elements are the ones most resistant to injurious and putrefactive events and are therefore used for forensic identification assessments in cases of doubt, assisted by other evidence if available. This is why DISs also acquire considerable importance from a forensic identification perspective. Indeed, properly positioned DIS can withstand thermal effects, allowing identification even in cases of complete body incineration. Naturally, in the case of laser use, the choice of technique is also crucial, requiring adequate irradiation power without altering the implant surface [36]. The knowledge of the most suitable materials and prosthetic techniques for this purpose is, therefore, a fundamental part of large-scale use. 

Therefore, it is necessary to compare the mentioned types of dental identification systems based on their functionality, applications, and benefits.

- Microchip Implants

Implanted microchips store unique identification information that can be read using a scanner. These are embedded in dental prostheses or teeth and can provide rapid identification in forensic cases. Offers permanent and tamper-proof identification, with information easily retrievable by authorities. They may involve higher initial costs and require specialized equipment for reading the data.

b. Prosthetic Inscriptions

Identifying marks or codes are engraved directly onto dental prostheses. Used primarily in dentures or other removable prostheses, making it easier to match them with the rightful owner in forensic or clinical scenarios. They are cost-effective and simple to implement, requiring no advanced technology. The inscription may wear out over time, and identification relies on the presence of the prosthesis.

c. MicroSD Cards

Small storage devices are embedded within dental prosthetics, containing detailed personal and medical information. They are useful in both forensic identification and clinical settings for accessing a patient’s medical history. They allow for the storage of large amounts of data, including dental records and personal identification. They may be more susceptible to damage, and the data retrieval process requires compatible technology.

d. Identification Plaques

Small plaques with encoded information are attached to or embedded within dental structures. They can be used similarly to prosthetic inscriptions but may provide more durable or detailed information. They offer a balance between simplicity and data capacity, potentially more durable than inscriptions. They are still relatively new, may have limited adoption, and could be overlooked during standard examinations.

Among these types of dental identification systems, prosthetic inscriptions are the most cost-effective, followed by identification plaques. Microchip implants and microSD cards are more expensive due to the technology involved but offer greater data capacity and reliability. Microchip implants and microSD cards excel in accuracy and speed, providing instant access to identification data. Plaques and inscriptions offer a simpler, though potentially slower, identification process.

In our opinion, the universal adoption of these systems could improve data exchange and healthcare outcomes, especially in vulnerable populations. Microchip implants and microSD cards would be particularly beneficial in clinical settings where detailed medical records need to be accessed quickly. In addition, all DIS types enhance forensic identification processes. However, microchip implants and microSD cards offer superior benefits due to their ability to store comprehensive data.

In conclusion, while each type of DIS has its strengths and weaknesses, their integration into forensic and clinical practices could significantly improve identification accuracy, streamline processes, and offer benefits across various sectors, particularly for vulnerable communities.

In Europe, as we have seen, although the marking of prostheses is encouraged primarily for traceability purposes, there appear to be some hindering factors regarding the widespread use and adoption of dental identification systems (DISs). However, the use of DISs in Europe could bring benefits both in clinical settings if applied to various health conditions and for their positive impact on personal identification processes, potentially leading to a reduction in associated expenses. Yet, the lack of utilization or limited use of DIS in Europe seems not solely attributed to the free and informed choice of individuals undergoing dental procedures. It is plausible to assume that the additional cost demanded by various professionals for implementing DISs, which is not mandated by law and thus borne by the citizen, along with the limited awareness and dissemination of the benefits for the patient, are the main obstacles to their widespread adoption [8,34]. In contrast, it has been observed that in parts of the US and India, dental prostheses are easily customizable using new technologies and low-cost thermoresistant materials [8,29]. Therefore, despite the strong recommendation for the use of DIS [35], it is evident that there is still much work to be performed to facilitate their adoption and dissemination; patient communication and information thus appear to play a prominent role. 

Furthermore, considering dentistry as a scientific field characterized by the use of industrially produced prostheses or custom-made ones, ensuring maximum quality and reliability in both work processes would be beneficial, perhaps through a European law indicating appropriate methodologies. Until then, although ensuring high-quality standards on dental prosthetic devices with DISs is inherently complex, having personal and clinical information along with the practitioner’s or hospital’s details embedded in a single prosthesis would provide significant assurance for the patient [37]. Including the information of the dentist or the hospital where the patient received dental care within DISs could be a useful tool for all patients to use in cases of medical malpractice. It is noteworthy that in some judicial systems, the burden of proof in cases of medical malpractice lies with the patient who has suffered harm rather than with the professional. In such cases, having DISs with personal medical information could serve as evidence when there are no other documents confirming the care relationship. Finally, as already specified, the forensic value of DISs could greatly simplify the work of forensic experts in cases of personal identification, even in the event of lethal incidents within the European territory. Considering the exchange of goods, people, and medical devices at the European and international levels and considering the regulations regarding the implementation of a European health data-sharing bank, the dissemination of the use of DISs could be a valuable aid in cases of dental treatments carried out outside the home country but within Europe, even solely to better ensure continuity of care within the community. 

Finally, it could also be considered to use dental identification systems (DISs) on a large scale for certain defined at-risk populations. These populations could include individuals whose lifestyle, hobbies, or work activities predispose them to situations that endanger their health and lives. For example, DISs could be employed in the Armed Forces and in law enforcement agencies, including healthcare personnel, who are daily committed to upholding laws and ensuring the safety of citizens. 

These individuals engaged in state security are exposed to numerous risks every day [38,39,40,41,42,43,44] risks, such as terrorist attacks, natural disasters, emergency situations, missions abroad (peacekeeping or wartime), exposure to carcinogenic substances (asbestos, fiberglass, and specific vaccinations for military personnel), or toxic and lethal substances (thorium and nerve gases). The daily dangers faced by law enforcement personnel can also lead to situations where emergency medical intervention is required, and clinical information may not always be readily available. Moreover, there may be situations where the identification of an unidentified body is necessary, both within national territory and outside one’s own country. This is why the military sector seems to represent the most suitable population for an initial assessment of the validity and usefulness of the use and dissemination of DIS. Utilizing DIS within the military could potentially address these challenges by providing rapid and accurate identification of individuals in emergency situations, enhancing medical response capabilities, and ensuring the prompt availability of vital medical information. Therefore, implementing DISs within military settings could serve as a pilot program to evaluate its effectiveness and practicality before broader implementation across other at-risk populations or sectors.

Although of great ethical and social importance, the current evidence on the development of DIS and the orientations in scientific research is oriented towards the adult population who could require implant-prosthetic rehabilitation. Over time, there remains the need to bridge this approach to modern forensic dentistry for identification purposes by including the child and adult population. In this sense, orthodontics still represents the specialist dental branch of reference for personal identification in the non-prosthetic population, and the integration of DISs in customized orthodontic appliances can be envisaged on who, due to oral conditions, cannot reasonably benefit from a prosthetically designed DIS. 

DIS can also be used for the exchange of health data in cases of individuals in vulnerable conditions. Vulnerable populations can be classified based on their vulnerability, which can be physical (women, older people, infants, and disabled people), psychological (individuals with mental health disorders or a history of alcohol and/or drug abuse) [45,46,47,48,49,50] or social (individuals living in disadvantaged family environments, people experiencing homelessness, immigrants, LGBTQ individuals, and certain minorities) [51,52]. In these cases, healthcare presents various ethical, forensic, and legal challenges. Often, individuals within these populations find themselves in precarious health situations, with their fundamental rights violated, lacking access to basic care, or unable to provide an overall view or accurate information regarding their medical history when necessary.

In this context, DISs are of significant importance; indeed, the implantation of DISs in some vulnerable individuals could facilitate the exchange of clinical information and health data in situations where these individuals are unable to provide clear information about their medical history. For example, in the case of physical vulnerability, older or disabled people could benefit from prosthetic implants containing clinical information accessible to healthcare professionals when they are physically unable to communicate. In cases of psychological vulnerability due to drug abuse, DISs could allow for the understanding of clinical history and potential interactions with otherwise prescribable medications during a medical emergency. In the case of social vulnerability, such as migrants or political refugees, the use of a DIS would greatly simplify the procedures for exchanging accurate clinical information, thereby increasing the level of care. 

From an ethical perspective, the use of DISs could ensure the rights of vulnerable populations to participate in decisions regarding their health, allowing them, for example, to give clearer and more informed consent. It could ensure fairer and more appropriate access to medical care in any country where the individuals might be located. Finally, it would guarantee healthcare providers targeted and accurate medical actions, avoiding harm to patients or themselves [38,53,54,55,56]. 

DISs could also ensure the exchange of healthcare information useful for forensic and legal purposes, guaranteeing data confidentiality and security, and preventing abuses or privacy violations.

## 5. Limitations, Ethical and Moral Implications

The limitations of using these dental devices are primarily related to the costs, which should be kept as low as possible or covered by the state in countries that can afford it, and the guarantee of confidentiality in data sharing. It will be necessary to develop strict privacy regulations to protect patient data [57,58] and establish ethical guidelines for their use, ensuring that data are used in a responsible and respectful manner. However, the ethical and moral implications of using these devices go far beyond these initial considerations. One of the main ethical aspects concerns the confidentiality and privacy of patient data. It is essential to develop stringent privacy regulations to protect citizens’ sensitive data, ensuring that it is used in a responsible and respectful manner. This includes adopting advanced security measures, such as those suggested by Europe and implemented in the USA and India, to prevent unauthorized access, data breaches, and misuse of personal information, especially in light of new technologies and artificial intelligence [59,60,61,62]. Another crucial point is informed consent. Patients must be fully aware of how their clinical data will be used and must give their explicit consent before any information is collected or shared. This process must be transparent and clear, without any parts difficult to understand for non-experts, clearly explaining the purposes of data use, potential risks, and benefits [63,64]. Another important moral consideration is that access to such devices should be equitable. Welfare policies should be developed to ensure that all citizens, regardless of their socioeconomic status, have access to these technologies. Otherwise, there could be disparities in the assistance and protection offered to different groups of the population. Moreover, the responsible use of the collected data is fundamental. This means that the information must be used exclusively for declared and legitimate purposes, such as identifying individuals in emergency situations or for medical purposes. The use of data for commercial, discriminatory, or undeclared purposes is ethically unacceptable and should be similarly unacceptable under international laws. Finally, the use of dental devices for identification also raises moral dilemmas concerning the surveillance and control of citizens, especially in some countries where fundamental citizen rights are not respected or even where authoritarian regimes are present. There is a risk that these technologies could be used to invade individual privacy, monitoring or profiling citizens without their consent. It is, therefore, essential to balance the need for security with respect for individual rights. In conclusion, while dental devices for identification offer significant potential benefits, it is crucial to carefully address the ethical and moral implications. Only through solid regulations, transparency, and responsible use can we ensure that these technologies are used in a way that respects the dignity and rights of individuals. Moreover, healthcare providers will need to be trained, and continuous technical support that is simple and low-cost must be ensured for the management and maintenance of the DISs. Finally, despite the potential of dental identification systems (DISs) to enhance the accuracy and speed of forensic identification, their use may be limited in certain contexts, particularly when integrated into fixed restorations or crowns. Our study indicates that most DIS devices appear to be designed for removable prosthetics, which may not be a fully viable option for those who utilize fixed restorations. Additionally, high-risk groups such as military personnel and firefighters, who are often exposed to extreme conditions, may not fully benefit from these technologies without further extensive and adequate studies. In such cases, DIS might be applicable only to natural teeth. The potential for device damage or destruction in complex operational environments reduces the effectiveness of their application for these categories.

## 6. Conclusions

Dental identification systems are crucial in forensic dentistry for identifying individuals and providing immediate results, unlike other methods such as DNA or radiographic examinations. This rapid identification is especially crucial in mass disasters. Dental elements, being highly resistant to damage and decay, are used for identification when other methods fail. DISs can withstand extreme conditions like incineration, making them essential in forensic contexts. Effective use of DISs relies on knowledge of appropriate materials and prosthetic techniques, highlighting the importance of expertise for their large-scale application. Indeed, ensuring high-quality standards for dental prosthetic devices with DISs is complex, but embedding personal, clinical, and practitioner or hospital information in a single prosthesis would greatly benefit patients and healthcare professionals.

From an ethical standpoint, the implementation of DISs must balance effective identification with privacy, ensuring strict data protection and informed patient consent, especially when used in forensic contexts. Additionally, a cost–benefit analysis is crucial, weighing the clear benefits of rapid identification against the costs of development and maintenance to ensure that a DIS is both ethically sound and economically viable.

DISs could serve as evidence in medical malpractice cases, especially in judicial systems where the burden of proof lies with the patient, and also in cases where healthcare professionals lack other evidence to prove their non-involvement in harmful events affecting the patient. DISs can also represent an integrated approach to addressing healthcare challenges for vulnerable populations. This requires a combination of technological innovation, social and ethical policies, and medical professionalism. DISs can thus play a crucial role in improving the quality of care, protecting patient rights, and facilitating more efficient and secure management of clinical information.

## Figures and Tables

**Table 1:** Overview of immediate dental identification systems.

Article (Ref.)	Country	DIS	Material	Manufacturing Method	Type of Patient
[20]	India	QR-code with Aadhar number	Metal prosthesis	Laser engraving	Alzheimer’s Disease
[21]	India	Barcode with patient information	Denture	Printing	Elderly and hospitalized patients
[22]	India	Barcoding with name, gender, age, address, phone number, and medical history	Denture	Application of the code in a 1 mm deep recess carved in the denture	Parkinson’s Disease
[23]	India	MicroSD with information costing 150 rupees (about 1.60 euros)	Denture	Integration of MicroSD into acrylic prosthesis	Unspecified
[24]	Chile	QR-code or MicroSD	Denture	Incorporation into denture material	Edentulous individuals
[25]	India	QR-code with Aadhar number	Denture	Incorporation into denture material	Patients in healthcare facilities, nursing homes, hospitals, and for medicolegal purposes
[26]	India	Labeling	Denture	Printing on heat-resistant polymer	Identification of individuals who have lost memory or have been involved in disasters
[8]	UK and US	Small paper card with name and hospital identification code in 89% of total cases; metal inclusion in 18% of US hospitals	Denture	Incorporation into denture	Cases required by law or at the discretion of dentists
[27]	India	Metal labeling systems	Denture	Incorporation	Patients with partial or complete edentulism for identification purposes
[28]	India	Memory card	Denture	Incorporation	Patients for identification purposes
[29]	India	Metallic QR code with patient details such as name, age, gender, address, medical history, and Aadhar number	Denture	Laser engraving of metallic QR code and incorporation into denture polymer	Edentulous patients for identification purposes
[30]	India	Barcode	Denture	Incorporation	To identify elderly, unconscious, or mass disaster-involved individuals
[31]	India and Saudi Arabia	Identification number made of metal	Denture	Incorporation	Edentulous patients for identification purposes
[32]	India	Barcode and unique identification number	Denture	Incorporation	To identify elderly, neurodegenerative disease patients, unconscious, or mass disaster-involved individuals
[33]	Australia	Individual numbers instead of batch serial numbers	Dental implants in heat-resistant metal (titanium and zirconium)	Laser engraving during production	Patients who have lost one or more teeth.
[6]	India	Personal identification code	Any type of fixed or removable prosthesis	Laser pen marking	Patients undergoing dental procedures for identification purposes.
[34]	UK	Identification code—metal label marking at a cost of 5–10 pounds per patient.	Any type of dental prosthesis	Incorporation into resin/polymer	Patient’s free choice to identify prosthesis in case of loss and oneself in case of fatal accidents.

## Data Availability

The data presented in this study are available from the corresponding author upon reasonable request.
